# Mapping Floral Genetic Architecture in *Prunus mume*, an Ornamental Woody Plant

**DOI:** 10.3389/fpls.2022.828579

**Published:** 2022-02-08

**Authors:** Mingyu Li, Mengmeng Sang, Zhenying Wen, Juan Meng, Tangren Cheng, Qixiang Zhang, Lidan Sun

**Affiliations:** ^1^Beijing Key Laboratory of Ornamental Plants Germplasm Innovation and Molecular Breeding, National Engineering Research Center for Floriculture, Beijing Laboratory of Urban and Rural Ecological Environment, School of Landscape Architecture, Beijing Forestry University, Beijing, China; ^2^Center for Computational Biology, College of Biological Sciences and Technology, Beijing Forestry University, Beijing, China; ^3^School Medicine, Institute of Reproductive Medicine, Nantong University, Nantong, China

**Keywords:** genetic mapping, floral trait, QTL, pleiotropy, transcriptomic analysis, *Prunus mume* (mei)

## Abstract

Floral traits are both evolutionarily and economically relevant for ornamental plants. However, their underlying genetic architecture, especially in woody ornamental plants, is still poorly understood. We perform mapping experiments aimed at identifying specific quantitative trait loci (QTLs) that control the size, shape, architecture, color, and timing of flowers in mei (*Prunus mume*). We find that the narrow region of chromosome 1 (5–15 Mb) contains a number of floral QTLs. Most QTLs detected from this mapping study are annotated to candidate genes that regulate various biological functions toward the floral formation. We identify strong pleiotropic control on different aspects of flower morphology (including shape, petal number, pistil number, petal color, and calyx color) and flower timing, but find different genetic systems that mediate whether a flower produces pistils and how many pistils a flower produces. We find that many floral QTLs display pleiotropic effects on shoot length growth but shoot radial growth, implicating a possible association of floral display with light capture. We conduct a transcriptomic study to characterize the genomic signature of floral QTLs expressed in mei. Our mapping results about the genetic control of floral features make it promising to select superior varieties for mei carrying flowers of ornamental value.

## Introduction

Floral traits play an important role in plant diversity, plant evolution, and ornamental plant breeding (Bradshaw et al., 1995; Ashman and Majetic, 2006; Ellis et al., 2014; Noman et al., 2017; Feng et al., 2019; Itagaki et al., 2020). In the ornamental plant industry, primary targets of selection and breeding for new plant varieties are elite floral traits with desirable anatomical attributes, shape, color, and time of flowering (Chandler and Sanchez, 2012; Azadi et al., 2016). The success to breed for favorable flowers is critically relied on how deeply we understand the genetic control of floral traits. For perennial woody plants, characterized by a large size, a long generation interval, and high heterozygosity and with a limited number of mutations, it is difficult or even impossible to generate inbred lines, as genetic resources appropriate for traditional quantitative and molecular genetic approaches (Aranzana et al., 2019). Because of this limitation, floral genetics for woody plants has largely lagged behind that in model systems and crop plants (Andres and Coupland, 2012; Pajoro, 2014; Chen et al., 2018).

Current genotyping techniques have steadily changed this situation by genotyping genome-wide single nucleotide polymorphism (SNP) markers for heterozygous woody plants at unprecedented resolution (Bielenberg et al., 2015; Santos et al., 2017; Aranzana et al., 2019; Zhao et al., 2019). By constructing genetic linkage maps from these markers, specific genetic factors, known as quantitative trait loci (QTLs), for phenotypic traits can be identified and mapped to narrow genomic regions. In the past decades, genetic mapping has been increasingly used as a routine approach to characterize QTLs for growth, branch, and leaf traits in ornamental woody plants (Sun et al., 2013, 2014, 2018; Zhang et al., 2015; Dong et al., 2017; Shi et al., 2020), shedding light on the genetic architecture of botanical architecture in trees. As reproductive traits, floral traits, such as floral size, shape, color, and timing, are likely to be under the control of polygenes (Cao et al., 2019; Feng et al., 2019; Yang et al., 2019; Chen et al., 2020). High-density genetic mapping allows these floral genes to be identified, their numbers, their genomic locations, their genetic effects, and their pleiotropic effects.

In this study, we conducted a genetic mapping study aimed to characterize QTLs responsible for floral traits in a woody ornamental plant, mei (*Prunus mume* Sieb. et Zucc.). Mei, originating in China, has been domesticated in East Asia for thousands of years (Chu, 1999). Mei possesses remarkable diversity and variation in flower size, morphology, architecture, and color, making it a favorable choice of ornamental plants in the floriculture industry. Since mei was sequenced (Zhang et al., 2012), considerable research has been carried out for the genetic dissection of botanical traits for this species, including stem growth, branch display, and heterophylly (Sun et al., 2013, 2014, 2018; Zhang et al., 2015; Jiang et al., 2019). In a recent genome-wide association study (GWAS) conducted by a set of 333 cultivars, Zhang et al. (2018) identified important QTLs for petal color, stigma color, calyx color, and bud color, some of which are located with the regions of candidate genes, such as v-myb avian myeloblastosis viral oncogene homolog 108 (*MYB108*). As a follow-up, we developed three linkage mapping populations by crossing morphologically contrast mei cultivars in the quest to systematically characterize flower QTLs and reveal their effects on floral size, anatomy, color, and timing. Results from genetic mapping, complementary to those from the previous GWAS, gain some new insight into the genetic architecture of floral traits in woody plants.

## Materials and Methods

### Plant Material

Three mapping populations used in this study were described in the previous study (Jiang et al., 2019), where an allometric model was developed to map growth QTLs in mei. Here, we focus on QTL mapping for floral traits. We crossed three pairs of mei cultivars that are in sharp contrast in terms of growth, branch structure, and floral traits to generate three full-sib families: F-2014 (*n* = 156) derived from Fenban (female) and Kouzi Yudie (male), L-2015 (*n* = 190) derived from Liuban (female) and Huang Lve (male), and Y-2015 (*n* = 184) derived from Liu Bandan (female) and Sanlun Yudie (male). We grafted scions from the seedlings of each family on 5-year rootstocks of healthy mei trees in the wintertime at the Experimental Station of Beijing Forestry University Center for Computational Biology, located in Nantong, Jiangsu Province, Southeast China. Scions sprout into shoots in the coming spring, which were cut off in the winter. Starting 2 years old, mei bloomed into flowers whose characteristics were measured. We randomly chose ten blooming locations of flowers on sprouts for each progeny to count their blossom buds. We record the date of blooming as the indicator of flower timing. After flowers grew into a full size, we randomly chose ten flowers from each genotype to count their petals and pistils, measure their floral diameters and pedicel lengths by using a slide caliper, record their floral shapes (shallow dish-shaped vs. bowl-shaped), and measure their petal color and calyx color by using a Pantone color card (color metrics from white to red).

### Single Nucleotide Polymorphism Genotyping

We obtained SNP genotype data by exacting DNA samples from young leaves. According to Sun et al. (2013), we extracted the SNPs with overall sequencing depths of more than 8, quality scores over 30, and at least four uniquely mapped reads per allele. After filtering, we obtained 1,484 segregating SNPs (261 testcross markers and 1,223 intercross markers) in the F-2014 population, 5,393 segregating SNPs (3,986 testcross markers and 1,407 intercross markers) in the Y-2015 population, and 5,012 segregating SNPs (4,477 testcross markers and 535 intercross markers) in the L-2015 population. There are two types of markers in a full-sib family derived from heterozygous parents: testcross markers at which one parent is heterozygous but the other is homozygous and intercross markers at which both the parents are heterozygous (Maliepaard et al., 1997; Wu et al., 2002; Lu et al., 2004; Tong et al., 2011).

### Analysis of QTL Location

Genetic linkage maps were constructed from testcross and intercross markers by JoinMap version 4 (Ooijen, 2006). We used the Kosambi map function to convert recombination fractions between markers to their map distances in centiMorgan (cM). The linkage map is composed of eight linkage groups paralleling to the haploid chromosome number of the mei genome. The total length of the map and the average intermarker distance are 750.85 and 0.51 cM in the F-2014 population, 714.85 and 0.11 cM in the Y-2015 population, and 919.85 and 0.17 cM in the L-2015 population, respectively.

We implemented a likelihood approach to estimate genotypic differences at individual SNPs in quantitatively-varying traits for each mapping population (Wu et al., 2007). The significance of genetic association for each SNP is tested by using a log-likelihood ratio (LR) test statistic. For quality traits, we used a logistic regression model to test the significance of SNPs. For both the quantitative and qualitative traits, the genome-wide critical thresholds are determined from empirical permutation tests. Because testcross markers and intercross markers have different degrees of freedom in the hypothesis test, we determined their critical thresholds, respectively (Wu et al., 2002; Lu et al., 2004). An SNP significantly associated with floral trait by permutation tests is called a QTL. Candidate genes linked with a QTL are determined by using a the basic local alignment search tool (BLAST).

### Differentially Expressed Genes Identification and Gene Annotations

We chose three different individuals for each genotype at a given floral QTLs to monitor gene expression profiles. Total RNA was extracted from the tissue by using TRIzol^®^ Reagent according to the instructions of manufacturer (Invitrogen, Carlsbad, CA, United States) and genomic DNA was removed by using DNase I (Takara Biotechnology Incorporation, Kyoto, Japan). Then, RNA quality was determined by using the 2100 Bioanalyzer System (Agilent Technologies, Santa Clara, CA, United States) and quantified by using the ND-2000 (NanoDrop Technologies, Wilmington, DE, United States). High-quality RNA sample (OD260/280 = 1.8–2.2, OD260/230 ≥ 2.0, RNA integrity number (RIN) ≥ 6.5, 28S:18S ≥ 1.0, and >10 μg) is used to construct sequencing library. RNA sequencing (RNA-seq) transcriptome libraries were prepared following the TruSeq™ RNA Sample Preparation Kit from Illumina (San Diego, CA, United States), by using 1 μg of total RNA. Shortly, messenger RNA (mRNA) was isolated with polyA selection by oligo (dT) beads and fragmented by using fragmentation buffer. Complementary DNA (cDNA) synthesis, end repair, A-base addition, and ligation of the Illumina-indexed adaptors were performed according to protocol by Illumina (San Diego, CA, United States). Libraries were then size selected for cDNA target fragments of 200–300 bp on 2% Low Range Ultra Agarose followed by PCR amplified by using Phusion DNA polymerase (New England Biolabs, Ipswich, MA, United State) for 15 PCR cycles. After being quantified by TBS380, paired-end libraries were sequenced by Illumina NovaSeq 6000 sequencing (150 bp × 2, Shanghai Biozeron Corporation Ltd., Shanghai, China). The raw paired-end reads were trimmed and quality controlled by Trimmomatic with parameters (SLIDINGWINDOW:4:15 MINLEN:75) (version 0.36).^1^ Then, clean reads were separately aligned to reference genome with orientation mode using hisat2^2^ software. This software was used to map with default parameters. The quality assessment of these data was taken by qualimap_v2.2.1.^3^ Use htseq^4^ to count each gene reads.

To identify DEGs between the two different samples, the expression level for each gene was calculated by using the fragments per kilobase of exon per million mapped reads (FRKM) method. R statistical package edgeR (empirical analysis of digital gene expression in R^5^) was used for differential expression analysis. The DEGs between two samples were selected by using the following criteria: the logarithmic of fold change was greater than 2 and the false discovery rate (FDR) should be less than 0.05. To understand the functions of the DEGs, the Gene Ontology (GO) functional enrichment and the Kyoto Encyclopedia of Genes and Genomes (KEGG) pathway analysis were carried out by Goatools^6^ and KEGG Orthology Based Annotation System (KOBAS).^7^ DEGs were significantly enriched in the GO terms and metabolic pathways when their Bonferroni-corrected *P*-value was less than 0.05.

## Results

**FIGURE 1:**
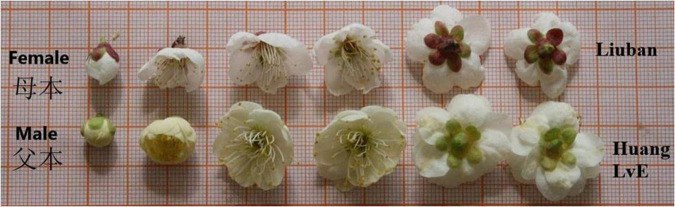
Differences in floral attributes between two parents, Liuban (female) and Huang LvE (male).

We constructed high-density linkage genetic maps that well cover the mei genome and mapped significant loci for floral traits to these maps for three full-sib families. We classify floral traits measured into five categories each capturing a different perspective of flower features: size (floral diameter and pedicel length), architecture (blossom bud number, petal number, and pistil number), shape, color, and timing. We identified a set of significant SNPs, called QTLs thereafter, associated with floral traits in three mapping populations. We describe our findings by using the L-2015 population. Two parents, female Liuban and male Huang LvE, for this population differ considerably in many flower features, including size, shape, petal color (white vs. light yellow), calyx color, and petal number among others (Figure 1). Results from all the three populations are compared to strengthen our overall conclusion about floral genetic architecture.

### Size QTLs

**FIGURE 2:**
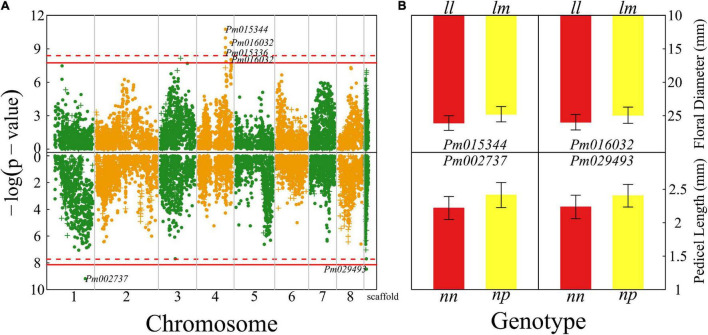
Identification of quantitative trait loci (QTLs) for flower size throughout the mei genome in a full-sib family of mei (L-2015 population) derived from Liuban (female) and Huang Lve (male) cultivars. **(A)** The Manhattan plots of significance test for floral diameter (upper panel) and pedicel length (lower panel). Solid and dashed lines represent the genome-wide critical thresholds of testcross and intercross markers, respectively, determined from 1,000 permutation tests. Single nucleotide polymorphisms (SNPs) annotated with biological functions are indicated. **(B)** Mean values (±SE) of different genotypes at two representative QTLs detected for floral diameter (upper panel) and pedicel length (lower panel).

Floral diameter and pedicel length reflect the size of a flower. We detected seven QTLs for floral diameter that are all located on chromosome 4 (Figure 2A) and two QTLs for pedicel length, with one detected on chromosome 1 and the other whose chromosome is unknown. There is no overlap of QTLs for floral diameter and pedicel length. Most of the QTLs for both the size traits are annotated to the genomic regions of candidate genes with known biological functions. For example, *Pm015344* is related to gene cyclic nucleotide-binding transporter 1 (*CNBT1*) that mediates calmodulin-binding, cyclic nucleotide-binding, and ion channel (Bridges et al., 2005). *Pm016032* is proximal to the gene coding K14396 polyadenylate-binding protein 2 and *Pm002737* is close to a Swi3, Ada2, N-Cor, and TFIIIB (SANT)-associated gene mediating many chromatin remodeling proteins to interact with histones (Horton et al., 2007).

We chose two representative QTLs for each trait to illustrate the specific effect of a QTL on floral size (Figure 2B). These chosen QTLs are testcross QTLs, i.e., those whose segregation in the full-sib family is only due to one heterozygous parent. The floral diameter of homozygote ll at *Pm015344* is thicker by 10% than that of heterozygote lm, whereas at *Pm016032* homozygote ll has a floral diameter wider by 9% than heterozygote lm. For two QTLs affecting pedicel length, *Pm002737* and *Pm029493*, heterozygotes have a longer pedicel (by 10–12%) than homozygotes.

### Shape QTLs

**FIGURE 3:**
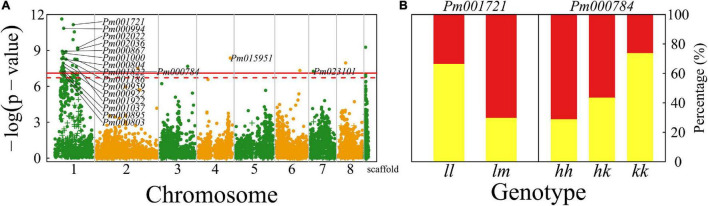
Identification of QTLs for flower shape throughout the mei genome in a full-sib family of mei (L-2015 population) derived from Liuban (female) and Huang Lve (male) cultivars. **(A)** The Manhattan plot of a significance test. Solid and dashed lines represent the genome-wide critical thresholds of testcross and intercross markers, respectively, determined from 1,000 permutation tests. SNPs annotated with biological functions are indicated. **(B)** Distribution percentages of shallow bowl-shaped flowers (red) and dish-shaped flowers (yellow) for the same genotype at two representative QTLs detected.

We identified a number of significant QTLs (103) for floral shape, of which 93 (>90%) are located in the 5 Mb-long interval of chromosome 1, six are sporadically distributed on chromosomes 2, 3, 4, 6, 7, and 8, and one has an unknown location (Figure 3A). A majority of QTLs are proximal to candidate genes that mediate different biological processes (Supplementary Table 1). For example, *Pm000804* is related to gene RING finger protein115/126 (*RNF115/126*) encoding E3 ubiquitin-protein ligase (Mazzucotelli et al., 2006) and *Pm001721* is associated with gene embryo defective 2733 (*EMB2733*)/enhanced silencing phenotype 3 (*ESP3*) that mediates ATP-dependent RNA helicase and K12813 pre-mRNA-splicing factor ATP-dependent RNA helicase DEAH-box helicase 16 (DHX16) (Herr et al., 2006).

By choosing two presentative QTLs, we elucidate how a QTL affects floral shape (Figure 3B). At testcross QTL *Pm001721*, the proportion of dish-shaped flowers is double for homozygote ll, compared with heterozygote lm. *Pm000784* is an intercross QTL whose segregation in the full-sib family is derived from both the heterozygous parents. At this QTL, homozygote kk with alleles derived from one parent has a noticeably larger proportion of dish-shaped flowers than homozygote ll with alleles from the alternative parent. The proportion of dish-shaped flowers for heterozygote hk with two alleles each from a different parent is intermediate between those for the two homozygotes, although it is closer to homozygote hh, suggesting that this QTL determines floral shape in a partially dominant manner.

### Architecture QTLs

**FIGURE 4:**
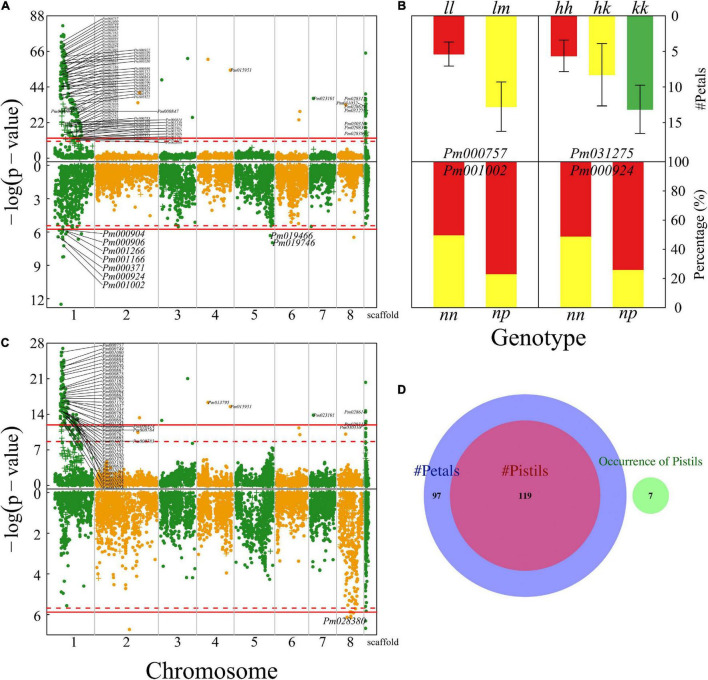
Identification of QTLs for floral architecture throughout the mei genome in a full-sib family of mei (L-2015 population) derived from Liuban (female) and Huang Lve (male) cultivars. **(A)** The Manhattan plots of significance test for petal number (upper panel) and blossom bud number (lower panel). Solid and dashed lines represent the genome-wide critical thresholds of testcross and intercross markers, respectively, determined from 1,000 permutation tests. SNPs annotated with biological functions are indicated. **(B)** Mean values (±SE) of different genotypes at two representative QTLs detected for petal number (upper panel) and distribution percentages of progeny with a single blossom bud (red) and multiple blossom buds (yellow) at two representative QTLs detected (lower panel). **(C)** The Manhattan plots of significance test for pistil number (upper panel) and the occurrence of pistils (lower panel). **(D)** The Venn diagram of QTL numbers for the number of petals, the number of pistils, and the occurrence of pistils.

The numbers of petals, pistils, and blossom buds are determinants of the architecture of a mei flower. We found numerous QTLs (324) that affect petal number and several QTLs for the number of blossom buds, a majority of which are collectively distributed in the 15 Mb-long interval of chromosome 1 (Figure 4A). A majority of QTLs detected are mapped to candidate genes based on the KEGG analysis (Supplementary Table 1). For example, *Pm000757* is related to the K11671 nuclear factor relevant to kappa-B-binding protein and *Pm031275* may have a function associated with K00423 L-ascorbate oxidase. We illustrate how these two QTLs influence the number of petals (Figure 4B). At testcross QTL *Pm000757*, heterozygote lm has more than double petals of homozygote ll, whereas intercross QTL *Pm031275* affects petal number in an additive manner. Two testcross QTLs *Pm001002* and *Pm000924* each have two genotypes that remarkably differ in the proportion of progeny with single blossom bud vs. multiple blossom buds (Figure 4B).

The number of pistils is a floral trait of interest to evolutionary studies and floriculture. Whether there is a pistil within a flower and how many pistils a flower carries are two different types of traits. The former is a quality trait, for which we used a logistic-regression model to map its QTLs, whereas the latter is a quantitative trait analyzed and mapped by a likelihood approach. We found 158 QTLs on chromosome 1 for pistil number, but only identified seven QTLs that determine the occurrence of pistils, none of which is located on chromosome 1 (Figure 4C). We found that all the QTLs for pistil number are part of the QTLs for petal number, indicating that these two architectural traits may be pleiotropically highly correlated (Figure 4D). However, we did not find any common QTLs for the occurrence of pistils and their number, from which we suggest that different genetic systems control whether a flower grows pistils and how many pistils a flower grows. The first process behaves like a switch, controlled by a few QTLs, whereas the second process is more complex, involving numerous QTLs.

### Color QTLs

**FIGURE 5:**
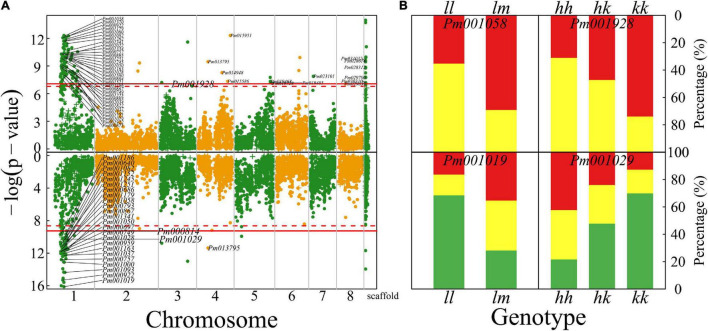
Identification of QTLs for petal color and flower timing throughout the mei genome in a full-sib family of mei (L-2015 population) derived from Liuban (female) and Huang Lve (male) cultivars. **(A)** The Manhattan plots of significance test for petal color (upper panel) and flower timing (lower panel). Solid and dashed lines represent the genome-wide critical thresholds of testcross and intercross markers, respectively, determined from 1,000 permutation tests. SNPs annotated with biological functions are indicated. **(B)** Distribution percentages of yellow flowers (red) and white flowers (yellow) for the same genotype at two representative QTLs detected for petal color (upper panel) and distribution percentages of early- (red), middle- (yellow), and late-blossomed flowers (green) for the same genotype at two representative QTLs detected for flower timing.

A total of 173 QTLs were identified for petal color; especially, there is a strong signal on chromosome 1 (137 QTLs) for this trait (upper panel, Figure 5A). Several QTLs for petal color are distributed on the other chromosomes, some of which on chromosome 4 are very close to those detected by our previous GWAS (Zhang et al., 2018). Two parents have different calyx colors, green for Liuban and yellow-white for Huang LvE (Figure 1), whose 190 full-sibs display a strong deviation of segregation with 1 green calyx and 189 yellow-white calyxes (data not shown). A larger sample size is needed to reasonably map the QTLs for calyx color. A majority of color QTLs can be annotated to candidate genes (Supplementary Table 1). For example, *Pm001058* is close to gene *K03453* mediating bile acid sodium symporter (BASS) family proteins. This is a testcross QTL whose homozygote ll and heterozygote lm tend to have much more white petals and yellow petals, respectively (upper panel, Figure 5B). *Pm001928* is related to gene *K12586 for* exosome complex component ribosomal RNA processing protein 43 (RRP43) proteins. As an intercross QTL, homozygote hh, heterozygote hk, and homozygote kk have about 25, 50, and 70% of petals that are yellow (upper panel, Figure 5B), suggesting its additive inheritance model for petal color.

### Timing QTLs

Plants have evolved to flower at a time of year to maximize their reproductive success in a given region. We identify 104 QTLs for mediating flower timing in mei, which are mostly distributed on chromosome 1, with only a few located on other chromosomes (lower panel, Figure 5A). A majority of timing QTLs detected are proximal to genes that have various biological functions (Supplementary Table 1). *Pm001019*, residing in the gene encoding RNA methyltransferase, is a testcross QTL. About 75% of progeny for homozygote ll at this QTL are late blossomed, whereas the rest are half early blossomed and half intermediately blossomed (lower panel, Figure 5B). At intercross QTL *Pm001029*, homozygote kk contributes to almost a half of late-blossomed flowers, and heterozygote hk and the alternative homozygote jointly contribute to the other half (lower panel, Figure 5B). Over 40% of progeny for homozygote hh are early blossomed, whereas this value is 15 and 25% for homozygote kk and heterozygote hk, respectively.

### Pleiotropic QTLs for Reproductive and Vegetative Growth

**FIGURE 6:**
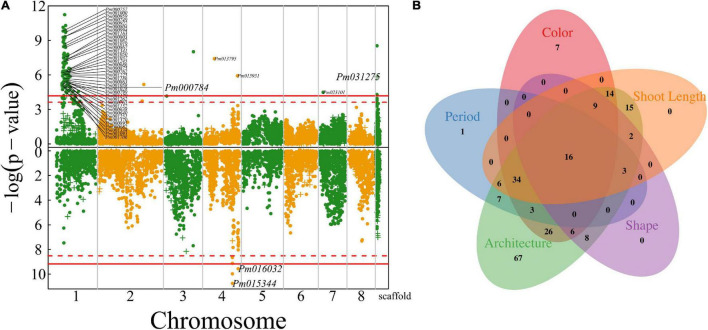
Identification of QTLs for shoot growth throughout the mei genome in a full-sib family of mei (L-2015 population) derived from Liuban (female) and Huang Lve (male) cultivars. **(A)** The Manhattan plots of significance test for shoot length growth (upper panel) and shoot diameter growth (lower panel). Solid and dashed lines represent the genome-wide critical thresholds of testcross and intercross markers, respectively, determined from 1,000 permutation tests. SNPs annotated with biological functions are indicated. **(B)** The Venn diagram of QTL numbers for shoot length, floral color, floral shape, floral architecture, and floral timing.

We further map QTLs for the length and diameter growth of shoots from which flowers are blossomed. Much more QTLs were detected to affect shoot length than shoot diameter growth (Figure 6A), i.e., 143 QTLs for the former and three QTLs for the latter. A majority of shoot length QTLs are distributed in the specific interval of chromosome 1 where floral QTLs are also located. Most shoot growth QTLs detected are highly related to candidate genes (Supplementary Table 1). Surprisingly, we did not identify any common QTLs that are shared between shoot growth (length and diameter) and floral size, but find a large pool of common QTLs for shoot length and floral architecture (described by blossom bud number, pistil number, and petal number), floral color, and flower timing (Figure 6B). Most of these common QTLs are located in a narrow region of chromosome 1, suggesting that this is an important pleiotropic segment that not only mediates shoot length growth, but also affects a number of floral traits. Shoot growth vigor is associated with reproductive life history in plants. However, it is unclear how vegetative growth is pleiotropically correlated with floral traits in mei. This study provides a new insight into the pleiotropic control of these two types of traits.

### Transcriptional Analysis of Floral QTLs

**FIGURE 7:**
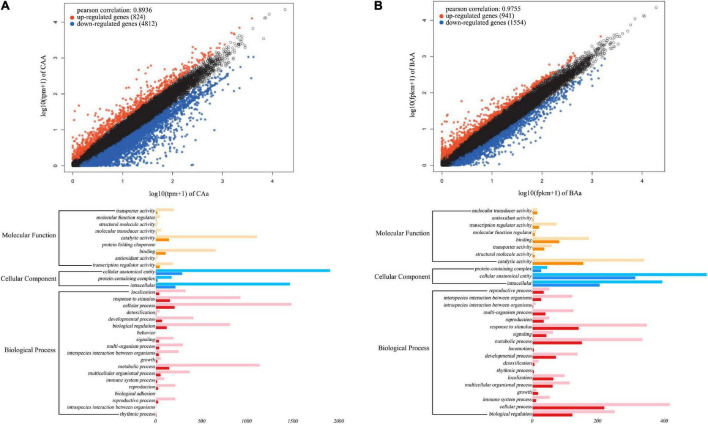
Transcriptomic signature of QTLs for petal color **(A)** and petal number **(B)** in the F-2014 population. **(Upper panel)** Differently expressed genes from genotype CAa to CAA for petal color and from genotype Baa to BAA for petal number. **(Lower panel)** The Gene Ontology (GO)-based gene enrichment analysis for genotypic differences for petal color and petal number.

To characterize the transcriptomic signature of floral QTLs detected, we test differentiated gene expression between different genotypes at a given QTL. Our analysis is focused on QTLs for petal color and petal number in the F-2014 population. Based on 15 petal color QTLs detected, we chose three purple-petal individuals (with consistent genotype CAA at each QTL) and three white-petal individuals (with consistent genotype CAa at each QTL). We find that 5,636 genes are expressed differently between two types of flower color, with 824 (15%) upregulated genes and 4,812 (85%) downregulated genes from purple flowers to white flowers (upper panel, Figure 7A). Similarly, at 15 petal number QTLs, double-petal genotype BAA and single-petal genotype BAa, each with three individuals, differ in gene expression. We find that of 2,526 genes, 941 (37%) and 1,584 (63%) genes are upregulated and downregulated from double petals to single petals (upper panel, Figure 7B). The GO gene-set enrichment analysis shows that these petal color- and petal number-related differentiated genes affect a wide spectrum of biological processes, but function in a narrow domain of molecular functions and, especially, cellular components (lower panel, Figure 7). The most numerous genes that determine petal color and number are those encoding cellular anatomical entities, followed by those encoding intracellular proteins, catalytic activity, cellular processes, metabolic processes, and response to stimuli.

**FIGURE 8:**
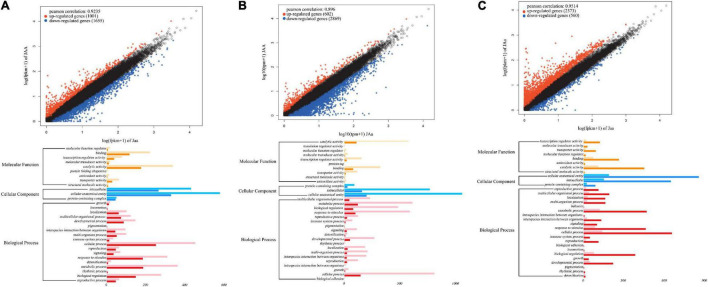
Transcriptomic signature of QTLs for petal diameter. Genomic comparison is made in terms of differentiated genes and their function between the two homozygotes **(A)**, between one homozygote and heterozygote **(B)**, and between the alternative homozygote and homozygote **(C)**.

Based on four floral diameter QTLs (intercross SNPs each with three genotypes), we chose three individuals for each genotype, JAA, JAa, and Jaa and compared and tested floral diameters among three genotypes (*P* < 0.05). Of 2,656 genes differentially expressed between the two homozygotes, 1,001 (38%) and 1,655 (62%) genes are upregulated and downregulated from Jaa to JAA, respectively (Figure 8A). There are 17–19 and 81–83% of genes that are upregulated and downregulated, respectively, from heterozygote Jaa to two homozygotes (Figures 8B,C). The GO analysis reveals the relative richness of functionally different genes that mediate petal diameter in mei (Figure 8B).

## Discussion

Flowers are morphologically complex traits. In this study, we use five key morphological attributes to systematically define a flower. These attributes, covering floral size, shape, architecture, color, and period, each describe a different feature of a flower. In nature, these floral traits determine the higher reproductive behavior and speciation of plants (Bradshaw et al., 1995; Bernardello et al., 2001) and play an important role in mediating the domestication of plants (Dubois et al., 2010). Like other complex traits, flower traits are most likely to be polygenic (Bradshaw et al., 1995; Galliot et al., 2006; Budahn et al., 2014; Zhang et al., 2018; Feng et al., 2019), although their overall genetic architecture still remains elusive.

In this study, we develop three genetic mapping populations (denoted as F-2014, L-2015, and Y-2015) by using different pairs of parents that differ in floral traits to characterize how specific QTLs govern floral traits in mei (*Prunus mume*). We focus on the interpretation of results from the L-2015 population. The most remarkable finding from this population is the discovery of a number of QTLs distributed in a small region of chromosome 1 (5–15 Mb) responsible for many floral traits (Figures 3–6). More interesting, this finding is broadly supported by the F-2014 population in which chromosome 1 was detected to be rich in floral QTLs (Supplementary Figure 1). In the Y-2015 population, we identified much fewer QTLs for floral traits (Supplementary Figure 2), suggesting that two parents used to produce this population are not largely heterozygous at floral QTLs, making the family less informative for QTL detection.

In a recent GWAS study by using 333 mei accessions, chromosomes 3 and 4 were found to harbor floral QTLs that control petal color, calyx color, and petal number (Zhang et al., 2018). Our mapping study broadly confirms the GWAS finding, but identifies a number of new floral QTLs. There may be several reasons that cause discrepancies between our high-density mapping and the previous GWAS. First, these two genetic approaches are complementary. While GWAS is powerful for finding a wide spectrum of natural genetic variants, genetic mapping can capture the segregation of rare alleles by crossing rare allele-carrying parents into a full-sib family. In other words, if variants due to rare alleles are important determinants of floral traits, genetic mapping is more advantageous over GWAS in identifying these variants. It is worthwhile to test whether QTLs detected in mapping populations but not in the GWAS population are rare-allele loci. Second, our mapping populations were planted in a different environment from that where GWAS cultivars are grown. Also, flowers sampled for GWAS and our mapping study were formed from different ages of trees; adult grafted trees (ages >50 years) for the former and young sprouted scions for the latter. Thus, it is possible that the environment and development have contributed to differences in QTL detection between the two studies.

We identify a set of QTLs that are shared among different attributes of mei flower. For example, all the QTLs for pistil number are found to be involved in controlling petal numbers, showing strong pleiotropic control over these two architectural traits. There are numerous QTLs that are common for petal color, calyx color, floral shape, and flowering period. Interestingly, these floral traits share a number of common QTLs with shoot length but shoot diameter, although the latter two traits are highly correlated. We interpret this to be due to the dependence of floral appearance and display on light. Relative to shorter shoots, taller shoots can capture more light, which then help the display of flowers. In practice, the identification of pleiotropic QTLs for vegetative growth and reproductive growth could be useful for marker-assisted breeding for superior mei cultivars with optimal tree form and floral traits. There has been a considerable body of literature on the detection of floral QTLs in a variety of plants species, especially annuals (Bradshaw et al., 1995; Andres and Coupland, 2012; Budahn et al., 2014; Ma et al., 2021). Yet, a systematic characterization of pleiotropic QTLs that affect multifaceted features of flowers, especially perennial flowers, is lacking. This study shed on the genetic architecture of floral traits in mei and other related species.

We identified a number of QTLs for floral traits within a narrow region of chromosome 1. For our controlled-cross populations, these QTLs may affect trait phenotypes through their true effects or due to their linkage with true floral QTLs. However, a majority of QTLs identified can be annotated to candidate genes that have various biological functions (Supplementary Tables 1–3), which may provide a starting point to characterize the precise molecular basis of genetic variation in floral traits. An in-depth mechanistic understanding of floral variation holds a great promise to transform genetic information into practical breeding schemes in mei, one of our most favorable ornamental plants.

## Data Availability Statement

The datasets presented in this study can be found in online repositories. The names of the repository/repositories and accession number(s) can be found below: https://www.ncbi.nlm.nih.gov/, PRJNA761529.

## Author Contributions

LS managed the project and wrote the manuscript. QZ and LS planned and coordinated the project. LS, ML, MS, ZW, and JM collected and grew the plant materials. LS, ZW, ML, and JM prepared samples. LS, ML, and MS conducted floral genetic architecture, QTL analysis, and transcriptome sequencing. All authors contributed to the article and approved the submitted version.

## Conflict of Interest

The authors declare that the research was conducted in the absence of any commercial or financial relationships that could be construed as a potential conflict of interest.

## Publisher’s Note

All claims expressed in this article are solely those of the authors and do not necessarily represent those of their affiliated organizations, or those of the publisher, the editors and the reviewers. Any product that may be evaluated in this article, or claim that may be made by its manufacturer, is not guaranteed or endorsed by the publisher.

## Footnotes

1
https://github.com/usadellab/Trimmomatic

2
https://ccb.jhu.edu/software/hisat2/index.shtml

3
http://qualimap.bioinfo.cipf.es/

4
https://htseq.readthedocs.io/en/release_0.11.1/

5
http://www.bioconductor.org/packages/release/bioc/html/edgeR.html/

6
https://github.com/tanghaibao/Goatools

7
http://kobas.cbi.pku.edu.cn/home.do
