# Supplementary material for: Mapping Floral Genetic Architecture in Prunus mume, an Ornamental Woody Plant
Source: Front Plant Sci. 2022 Feb 8;13:828579. doi: 10.3389/fpls.2022.828579 (PMC8860970; doi:10.3389/fpls.2022.828579)
Supplement: Supplementary file 1 [file Data_Sheet_1.DOCX]

Supplementary Material

# Supplementary Figures and Tables

## Supplementary Figures


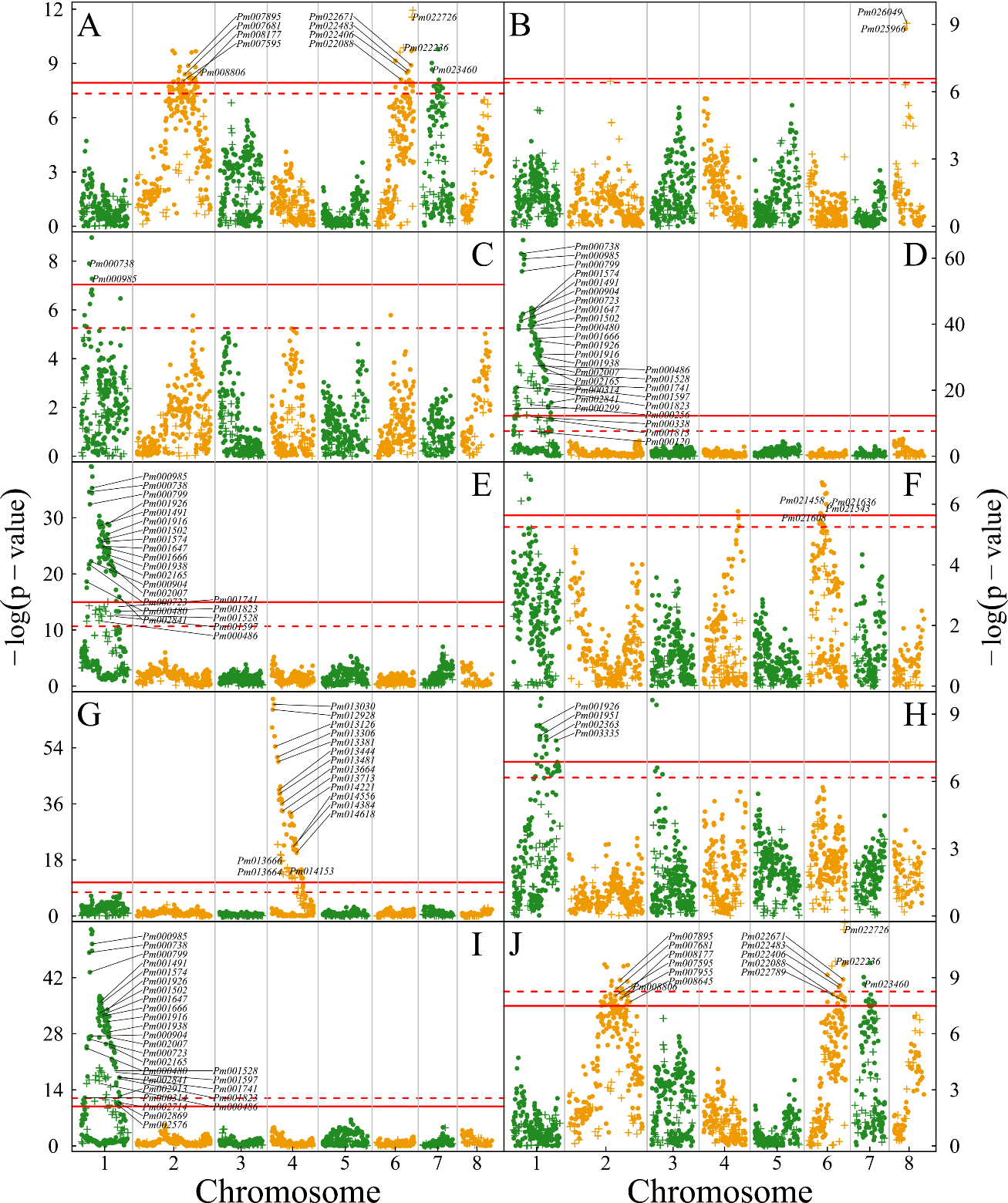


**Supplementary Figure 1.** **The Manhattan plots of significance test for floral diameter (A), pedicel length (B), floral shape (C), petal number (D), pistil number (E), blossom bud number (F), petal color (G), flower timing (H), shoot length (I) and shoot diameter (J) in the F-2014 population derived from Fenban (female) and KouziYudie (male) cultivars.** Solid and dashed lines represent the genome-wide critical thresholds of testcross and intercross markers, respectively, determined from 1000 permutation tests. SNPs annotated with biological functions are indicated.


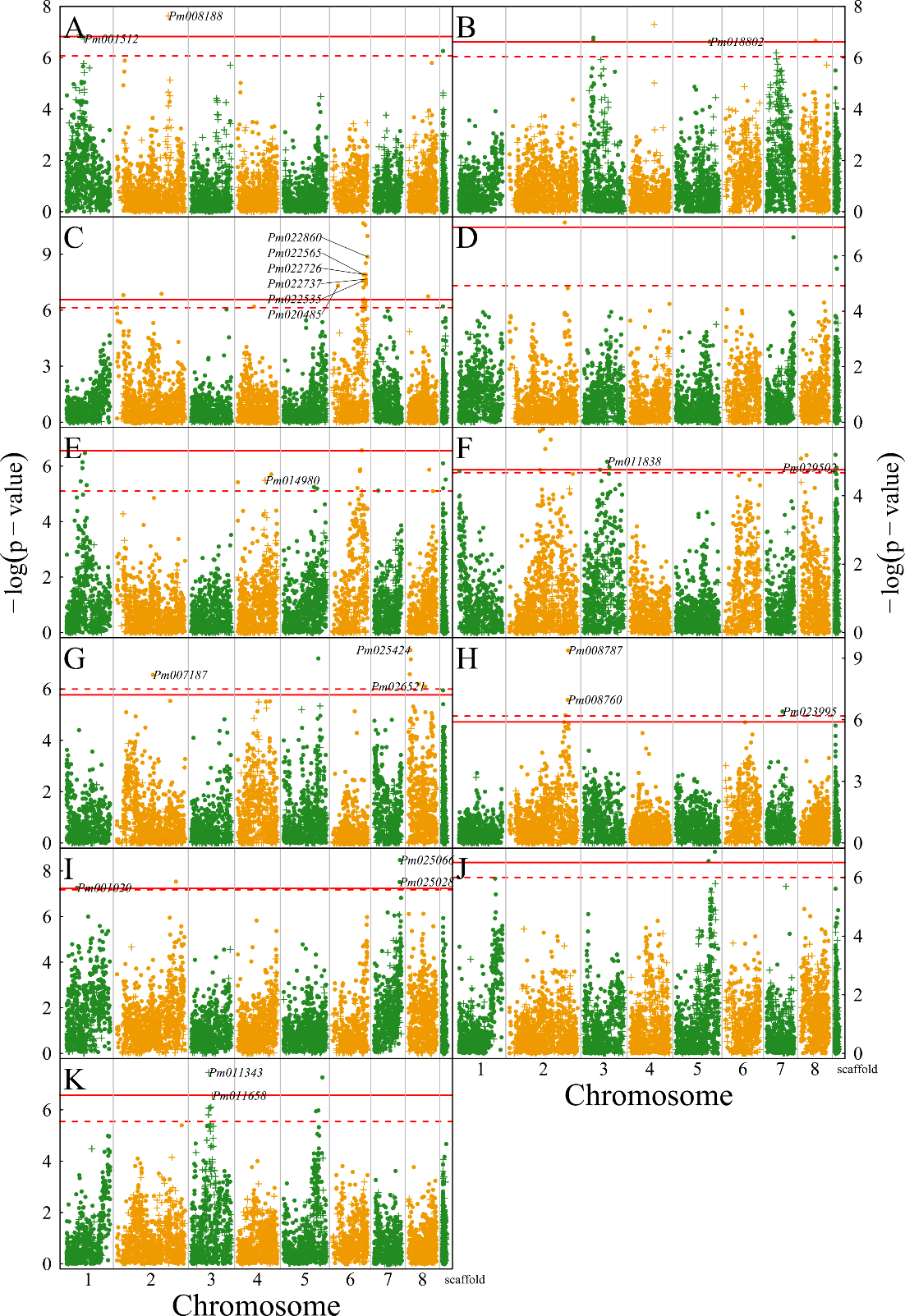


**Supplementary Figure 2.** **The Manhattan plots of significance test for floral diameter (A), pedicel length (B), floral shape (C), petal number (D), pistil number (E), blossom bud number (F), pistil occurrence (G), petal color (H), flower timing (I), shoot length (J) and shoot diameter (K) in the Y-2015 population derived from Liu Bandan (female) and SanlunYudie (male) cultivars.** Solid and dashed lines represent the genome-wide critical thresholds of testcross and intercross markers, respectively, determined from 1000 permutation tests. SNPs annotated with biological functions are indicated.

**Supplementary Table 1. Gene enrichment analysis of significant SNPs for floral traits in the L-2015 population derive from Liuban (female) and Huang Lve (male) cultivars.**

**Supplementary Table 2. Gene enrichment analysis of significant SNPs for floral traits in the F-2014 population derived from Fenban (female) and KouziYudie (male) cultivars.**

**Supplementary Table 3. Gene enrichment analysis of significant SNPs for floral traits in the Y-2015 population derived from Liu Bandan (female) and SanlunYudie (male) cultivars.**
